# Antibody-Dependent Cellular Cytotoxicity (ADCC)-Mediating Antibodies Constrain Neutralizing Antibody Escape Pathway

**DOI:** 10.3389/fimmu.2019.02875

**Published:** 2019-12-11

**Authors:** Dieter Mielke, Gama Bandawe, Justin Pollara, Melissa-Rose Abrahams, Tinashe Nyanhete, Penny L. Moore, Ruwayhida Thebus, Nicole L. Yates, John C. Kappes, Christina Ochsenbauer, Nigel Garrett, Salim Abdool Karim, Georgia D. Tomaras, David Montefiori, Lynn Morris, Guido Ferrari, Carolyn Williamson

**Affiliations:** ^1^Division of Medical Virology, Institute of Infectious Diseases and Molecular Medicine, University of Cape Town, Cape Town, South Africa; ^2^Duke University Medical Center, Durham, NC, United States; ^3^HIV Virology Section, Centre for HIV and STIs, National Institute for Communicable Diseases, Johannesburg, South Africa; ^4^National Health Laboratory Service, Johannesburg, South Africa; ^5^MRC Antibody Immunity Research Unit, University of Witwaterstrand, Johannesburg, South Africa; ^6^Centre for the AIDS Programme of Research in South Africa (CAPRISA), University of KwaZulu Natal, Durban, South Africa; ^7^Department of Medicine, University of Alabama at Birmingham, Birmingham, AL, United States; ^8^Research Service, Birmingham Veterans Affairs Medical Center, Birmingham, AL, United States; ^9^Discipline of Public Health Medicine, School of Nursing and Public Health, University of KwaZulu Natal, Durban, South Africa; ^10^Department of Epidemiology, Columbia University, New York, NY, United States

**Keywords:** ADCC, CD4-induced, neutralizing antibody, selection, escape

## Abstract

Both neutralization and antibody-dependent cellular cytotoxicity (ADCC) may be required for effective protection against HIV-1 infection. While there is extensive information on the targets of early neutralizing antibody (nAb) responses, much less is known about the targets of ADCC responses, which are more difficult to characterize. In four individuals recruited during acute HIV-infection, ADCC responses were detected 3–7 weeks prior to nAb responses. To determine the relative influence of ADCC and nAb responses on virus evolution, we performed an in-depth investigation of one individual (CAP63) who showed the highest nAb and ADCC responses. Both nAbs and ADCC antibodies targeted the V4 region of the Env, although there were some differences in epitope recognition. We identified accelerated viral evolution in this region concurrent with emergence of nAb activity, but not ADCC activity. Deep sequencing demonstrated that most nAb escape mutations were strongly selected for, however one nAb escape mutation that rendered the virus highly susceptible to autologous ADCC responses, was suppressed despite not affecting viral fitness. This escape mutation also rendered the virus more sensitive to autologous responses, as well as monoclonal antibodies targeting CD4-induced epitopes, compared to the wildtype virus. In conclusion, ADCC responses and nAbs in donor CAP63 recognized overlapping but unique epitopes in the V4 region, and while ADCC activity was present prior to nAbs, it did not drive viral evolution during this time. However, ADCC responses may select against nAb escape pathways that expose other common ADCC epitopes thereby restricting viral replication and expansion.

## Introduction

Both neutralization and effector cell functions, mediated through the antibody Fc domain, may be required for effective protection against HIV-1 infection (1). Neutralizing antibody (nAb) responses block viral entry into target cells by binding the HIV-1 envelope glycoprotein (Env) on infectious virions, while there are multiple Fc-mediated effector cell functions, including phagocytosis of opsonized virus particles or infected cells, antibody-dependent complement-mediated lysis, and stimulation of antiviral cytokines/chemokine secretion (2). The effector function antibody-dependent cellular cytotoxicity (ADCC), investigated in this study, mediates NK cell killing of HIV-1 infected cells through the binding of antigen-antibody complexes to the FcγRIIIa receptor (CD16) expressed on NK cells (3). These responses play a role in curbing early SIV viral replication (4–6), are enriched in human HIV-1 infected non-progressors (7), and may contribute to protection from HIV-1 infection, as suggested by the results of the HIV-1 RV144 vaccine trial (8–11). Furthermore, ADCC-mediating, non-neutralizing antibodies in breast milk have been correlated with reduced vertical transmission from viremic mothers (12). It is not known if ADCC responses play a role in the early control of HIV-1 infection, information that would be informative for vaccine design and antibody-based therapeutic studies.

The high mutation rate of HIV makes it possible to use patterns of genetic evolution to evaluate targets and kinetics of immune pressure, providing information on the role of antibodies in controlling viral populations *in vivo*. However, dissecting the possible role of ADCC antibodies during early stages of HIV-1 infection has proved challenging. While several studies have defined epitopes targeted by ADCC-mediating antibodies [reviewed by (13)], many overlap with neutralizing antibody epitopes, making it difficult to unravel ADCC responses and nAb immune pressure on the virus. Only one study to date, of individuals with chronic infection, has clearly demonstrated HIV-1 escape from ADCC (14). In addition, many methods used to define ADCC epitopes rely on targets coated with peptides or recombinant forms of monomeric Env (13), which do not fully reflect the epitope exposure of the HIV-1 Envelope on a virus infected cell. Therefore, infected cells expressing native Env trimers are preferred to accurately map escape from ADCC responses in natural infection (15).

Here, we investigated the kinetics of early neutralizing and ADCC responses in four HIV-1 subtype C infected individuals. In one individual (CAP63), who developed the earliest and strongest nAb and ADCC responses, we mapped Env targets of neutralizing and ADCC-mediating antibodies, using pseudovirus and infectious molecular clone (IMC)-based assays. Both of these responses targeted the V4 region of the Env, although there were some differences in epitope recognition. In particular, a six amino acid deletion in V4 conferred resistance to nAbs but created sensitivity to ADCC responses; specifically, autologous responses and monoclonal antibodies targeting CD4-induced (CD4i) epitopes. Next-generation sequencing revealed that this deletion was strongly selected against, despite not incurring a replication fitness cost to the virus. These data suggest that nAb escape pathways can be constrained by ADCC activity, illustrating a role of ADCC activity in controlling early replication and an interplay between the neutralizing and effector-mediated functions of early antibody responses.

## Results

### ADCC Responses Detected Early in Infection Correlate With HIV-Specific Binding Antibodies

We first explored the kinetics of binding antibody and ADCC development in four HIV-1 infected individuals (CAP45, CAP63, CAP210, and CAP239) recruited within 2 to 4 weeks post-infection (WPI), all of whom were infected by a single HIV-1 subtype C variant (16). Binding antibody responses against the subtype C protein 1086cΔ7 gp120 was detected using the BAMA assay and ADCC-mediating antibodies were detected using the ADCC-GTL assay, with 1086cΔ7 rgp120-coated CEM.NKR_CCR5_ cells as targets. Neutralizing responses were detected using autologous transmitted/Founder (T/F) Env pseudoviruses.

**Figure 1:**
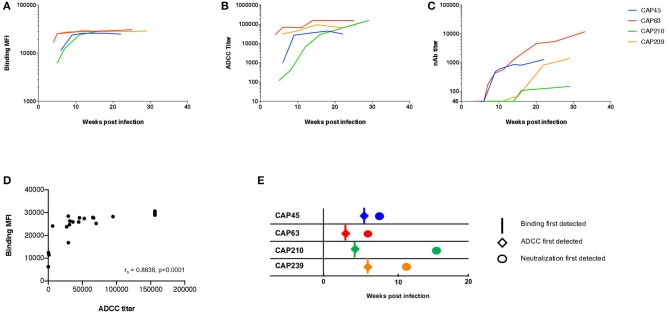
The kinetics of binding, ADCC, and nAb responses to HIV-1 subtype C in four participants of the CAPRISA 002 cohort. Binding (mean fluorescent intensity (MFI) at 1:50 dilution shown) of **(A)** and ADCC activity (titer, starting at 1:100 dilution) against **(B)** recombinant 1086cΔ7 gp120-coated CEM.NKR_CCR5_ cells, and the nAb responses against pseudoviruses with the T/F Envelope incorporated (ID_50_ titer, starting at a 1:45 dilution) **(C)** was determined longitudinally for four participants: CAP45 (blue), CAP63 (red), CAP210 (green) and CAP239 (orange). Binding MFI (at 1:50 dilution) and ADCC titer were plotted and a Spearman's r was calculated (*r*_s_ = 0.8838 (95% CI: 0.7359–0.9512), *p* < 0.0001) **(D)**. The time of first detection was determined for binding (vertical lines), ADCC (diamonds) and nAb (circles) responses **(E)**.

HIV-1 Env specific binding responses and ADCC responses were detected at the enrolment timepoint for all four individuals (Figures 1A,B). The nAb responses against the autologous transmitted/founder (T/F) Env emerged by 7, 9, 13, and 16 wpi in CAP63, CAP45, CAP239, and CAP210, respectively. For all individuals, autologous nAb responses were detected 3–7 weeks after binding responses and ADCC (Figures 1C,E). A strong association was detected between early binding responses and ADCC against the subtype C protein 1086cΔ7 gp120 (*r*_s_ = 0.884 (95% CI: 0.736–0.951), *p* < 0.0001; Figure 1D).

Previous studies suggest IgG1 and IgG3 are predominantly responsible for ADCC and neutralizing activities in HIV infection, with IgG3 levels declining within the first 6 months (17, 18). We investigated the HIV-specific IgG subclass antibody responses in one participant, CAP63, over the first 20 weeks of infection using an HIV-1 Binding Antibody Multiplex Assay (HIV-1 BAMA) against 17 envelope proteins (gp41/gp120/V1V2/gp140) of various clades and clade consensus sequences.

Moderate gp120/140 IgG1 specific responses were observed at 4 wpi, which increased by 20 wpi (Figure S1A). IgG1 bound most strongly to 1086C gp140 protein, with titers >1,000 at 4 wpi rising to 12,000 by 9 wpi. In comparison, binding of IgG3 to all Env proteins developed after IgG1 and at lower titers (Figure S1B). Both IgG1 and IgG3 HIV-1-specific antibodies were detectable at enrolment. As with other studies, gp41-binding antibody responses were observed at high titers at enrolment and maintained over 20 wpi, with gp41 IgG3 responses declining over the time period (Figures S1C,D) (18). V1/V2-specific binding antibody responses were detected after 13 wpi and only to one V1V2 scaffold (Figures S1E,F). No HIV-specific IgG2 and IgG4 binding was detected at enrolment or over the period studied (data not shown).

### Deletions in the V4 Are Prominent in CAP63 Viral Evolution

**Figure 2:**
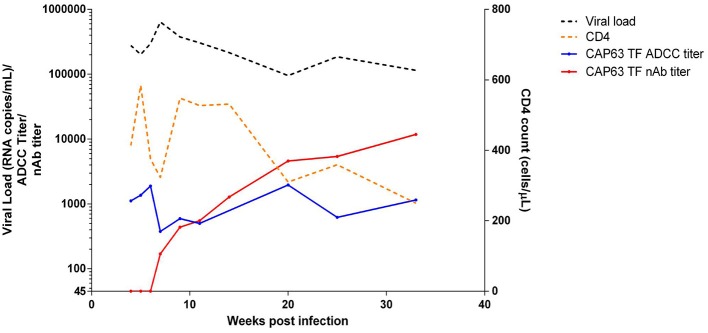
Clinical and humoral response profiles in one CAPRISA 002 participant: CAP63. Viral load (black), CD4 count (orange), ADCC-mediating antibody responses (titer, starting at 1:50 dilution) to CAP63 T/F IMC virus-infected CEM.NKR_CCR5_ cells (blue) and nAbs (ID_50_ titer, starting at 1:45 dilution) against CAP63 T/F pseudoviruses (red) were measured over 32 weeks of infection.

We then investigated CAP63, who had the earliest and strongest ADCC and nAb responses, in detail. This participant was identified after ~2 weeks of infection (HIV antibody negative and viral load positive) [Fiebig stage 1/2, (19)]. The participant experienced rapid disease progression, characterized by viral loads maintained above 100,000 RNA copies/ml and a rapid CD4+ T cell count decline over 32 weeks of infection (Figure 2) (19). Antiretroviral therapy was initiated at 37 wpi in accordance with prevailing South African Department of Health treatment guidelines.

**Figure 3:**
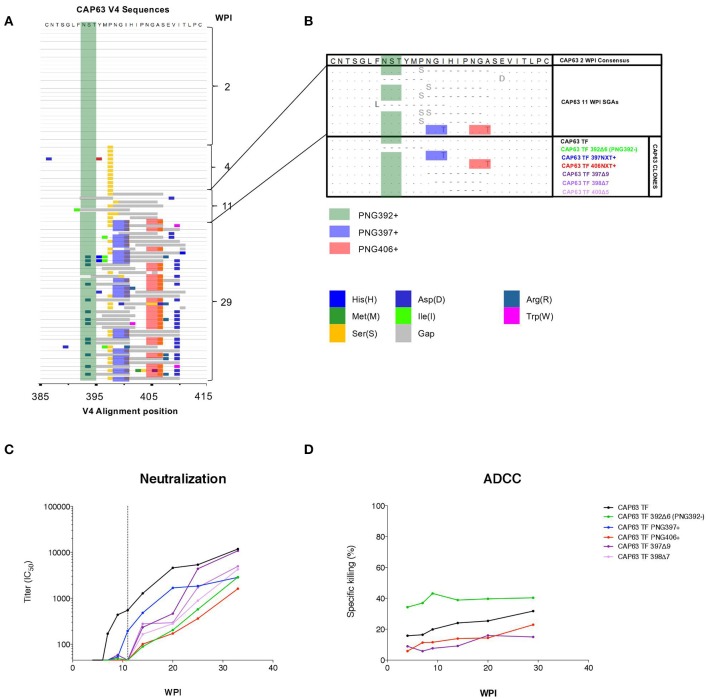
CAP63 V4 viral evolution and antibody escape. A highlighter plot was generated using 91 V4 sequences from 2 (*n* = 30), 4 (*n* = 11), 11 (*n* = 9), and 29 (*n* = 40) wpi **(A)**. Changes to the PNG profile at three sites are shown: at position 392 (dark green), 397 (blue), and 406 (red). Deletions are shown in gray. Naturally occurring changes in the V4 observed at 11 wpi (**B**, light blue) were then introduced as mutations in the T/F *env* and Infectious Molecular clones for use neutralization assays and ADCC assays (**B**, CAP63 clones). IMC constructs were used to infect CEM.NKR_CCR5_ target cells for used in an infected-cell elimination assay, while pseudoviruses were used in neutralization assays. The effect of each V4 change on neutralization sensitivity (ID_50;_ starting at 1:45 dilution; dotted line indicates 11 wpi) **(C)** and ADCC sensitivity (maximum levels of specific killing (%)) **(D)** was then determined. Figures show the mean of three independent experiments.

In order to investigate regions of the Env protein under antibody selection in CAP63, 90 full length gp160 sequences were generated from samples collected 2 (*n* = 30), 4 (*n* = 11), 11 (*n* = 9), and 29 (*n* = 40) wpi (Figure 3A). One mutation was observed in the V4 (P397S) in all 4 wpi sequences but not in the 20 sequences at 2 wpi indicating rapid selection of this residue. However, major shifts were observed after detection of nAbs at 7 weeks, and in particular in the V4, the known target of initial nAbs in this individual (20). By 11 wpi, seven of eight sequences contained deletions in the V4 region, while potential N-linked glycosylation site (PNG) shifts were observed throughout infection at three sites: PNG392 (dark green), PNG397 (blue), and PNG406 (red). By 29 wpi, all sequences had combinations of deletions and point mutations (including introductions of PNG sites at 397 and 406) in the V4.

### Changes in V4 Mediate Escape From Neutralizing and ADCC Antibody Responses

To map epitopes recognized by nAb and ADCC-mediating antibodies, we introduced naturally occurring changes observed at 11 wpi (Figure 3B) into functional CAP63 T/F *env* and T/F infectious molecular clones (IMCs) (Figure 3B, CAP63 clones). We used IMCs in the ADCC assays as these are more reliable than peptides or proteins for identifying conformational epitopes. Two point mutations in PNGs were introduced individually: I399T (PNG397+) and A408T (406NXT+). Further, four different deletions were introduced: a six-residue deletion from position 392, resulting in the loss of PNGs at 392 [392Δ6(PNG392-)], a nine-residue deletion from position 397 (397Δ9), a seven-residue deletion from position 398 (398Δ7) and a five-residue deletion from position 400 (400Δ5).

The T/F and mutant pseudoviruses were assayed against plasma from multiple time-points between 4 and 37 wpi. All the deletions and both the I399T and A408T mutant viruses (incorporating either PNG397 or PNG406, respectively) observed in the V4 loop resulted in either complete or partial resistance to nAbs at 11 wpi, after which nAbs against these viruses were detected (Figure 3C, shift of curves to the right). In comparison, the T/F IMC was sensitive to plasma ADCC-mediating antibodies from 4 to 29 wpi (Figure 3D). The 400Δ5, 397Δ9, and PNG406+ mutants were not recognized by ADCC-mediating antibodies at all time-points tested suggesting they enabled escape from ADCC responses. These data suggest that nAb escape mutations also mediate escape from ADCC-mediating antibodies.

Interestingly, however, the 392Δ6(PNG392–) mutation resulted in increased sensitivity (in comparison to the T/F virus) to ADCC-mediating antibodies at all the time points tested. This suggests that this deletion, which conferred resistance to nAbs, resulted in increased exposure of epitopes targeted by autologous ADCC-mediating antibodies.

### Deletion of the PNG392 (392Δ6) Increases Sensitivity to Antibodies Targeting CD4-Induced Epitopes

**Figure 4:**
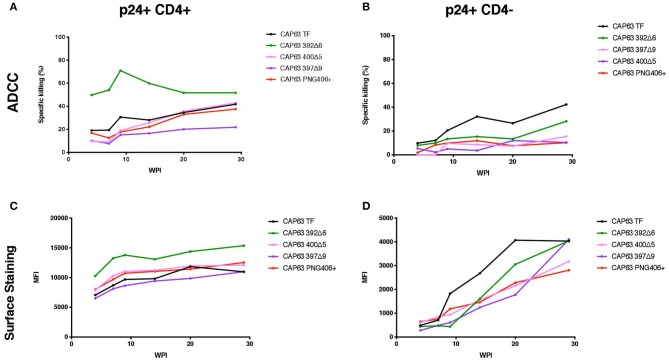
Autologous ADCC responses targeting, and surface staining of CD4+ and CD4– CAP63 IMC-infected CEM.KNR_CCR5_ cells. Longitudinal plasma was used to test the sensitivity of CD4+ **(A)** and CD4– **(B)** CAP63 IMC-infected cells to autologous ADCC responses and the ability of each antibody to bind to the surface of CD4+ **(C)** and CD4– **(D)** infected cells. ADCC activity is shown as the specific killing (%) by 1:100 dilution of plasma in an infected-cell elimination assay, and levels of surface binding are depicted by the median fluorescent intensity (MFI) of the secondary antibody. Figures show the mean of three independent experiments.

We sought to determine if the increased sensitivity of the 392Δ6(PNG392-) mutant to autologous ADCC was due to a change in the Env conformation which exposed otherwise hidden ADCC epitopes, such as the C1C2 epitopes. To do this, we first assessed the ability of autologous plasma to mediate ADCC against, and bind to the surface of, CAP63 virus-infected CD4 positive (CD4+) and negative (CD4-downregulated; CD4–) CEM.NKR_CCR5_ cells. Autologous ADCC responses against CD4+ infected cells, was higher against the 392Δ6(PNG392–) virus relative to the T/F (Figure 4A). In comparison, the three other mutants, (PNG406+, 397Δ9, and 400Δ5) were less susceptible to ADCC compared to the T/F virus. This observation was supported by surface staining, which showed plasma antibodies bound to the surface of 392Δ6(PNG392–)-infected cells more than the other variants (Figure 4C). In contrast, all the CAP63 mutants were resistant to ADCC and binding responses against infected cells with CD4 downregulated (CD4–), portraying a similar pattern to the neutralization profile (Figures 4B,D).

**Figure 5:**
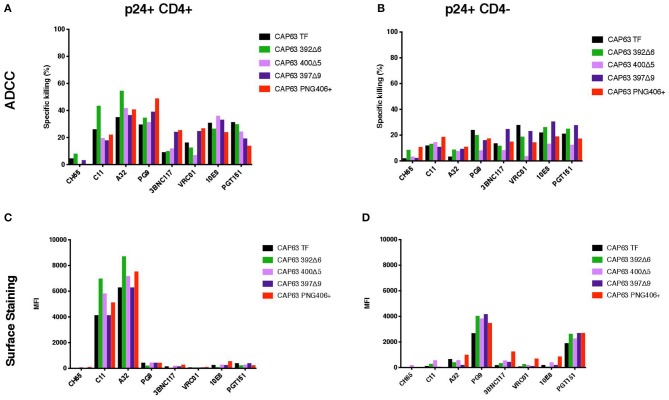
ADCC against, and surface staining of, CAP63 IMC-infected CEM.KNR_CCR5_ cells by a panel of anti-HIV-1 monoclonal antibodies. A panel of eight anti-HIV-1 monoclonal antibodies were used to test the sensitivity of CD4+ **(A)** and CD4– **(B)** CAP63 IMC-infected cells to ADCC and the ability of each antibody to bind to the surface of CD4+ **(C)** and CD4– **(D)** infected cells. ADCC activity is shown as the specific killing (%) by 10 μg/mL mAb in an infected-cell elimination assay, and levels of surface binding are depicted by the median fluorescent intensity (MFI) of the secondary antibody. Figures show the mean of three independent experiments.

We hypothesized that the increased sensitivity of the 392Δ6(PNG392–) virus was due to increased susceptibility to CD4i responses, as suggested by the enhanced killing of, and binding to, CD4+ cells. To determine if this mutation changed the structure to expose other common ADCC epitopes, we tested CD4+ infected cells against a panel of anti-HIV-1 mAbs [A32, C11 (CD4i epitopes); PGT151 (gp41-gp120 interface); 3BNC117, VRC01 (CD4 binding site); 10E8 (MPER), and PG9 (V2)]. All Abs had identical Fcs optimized to bind FcγRIIIa to mediate ADCC activity against cells infected with the T/F and mutant IMCs in the infected cell elimination assay and in infected cell surface binding assays (Figure 5). Of the seven mAbs we tested, only 392Δ6(PNG392-) showed increased susceptibility to C11 and A32 ADCC and binding (Figures 5A,C). These data suggest the ADCC^s^/nAb^r^ mutant (392Δ6[PNG392–]) alters the conformation of the Env in a manner that promoted recognition and binding by CD4i ADCC-mediating antibodies to 392Δ6(PNG392-) Env.

### Next-Generation Sequencing Reveals Strong Selection Against ADCC^s^/nAb^r^ Mutant 392Δ6(PNG392-)-Like Deletions

**Figure 6:**
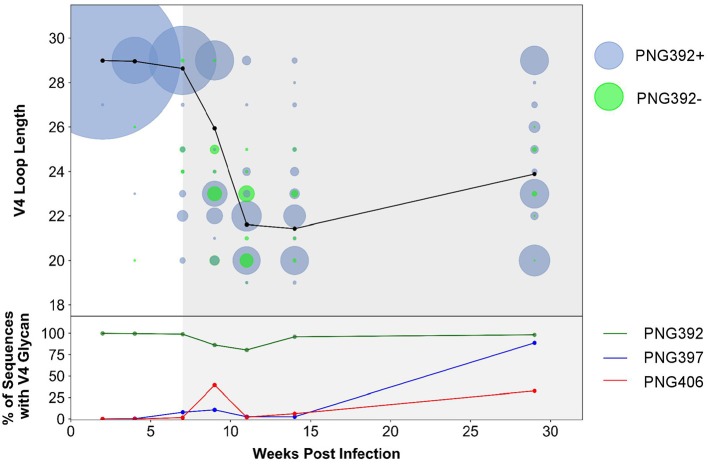
CAP63 V4 loop characteristics based on next-generation sequencing data. Next-generation sequencing of the V4 region at 2, 4, 7, 9, 11, 14, and 29 wpi was performed. The V4 loop length relative to HxB2 and the total number of viral copies at each loop length were calculated using a custom script **(top)**. Viral populations with the PNG392 present are shown in blue and viral populations with the 392NXT PNGS removed are shown in green. The size of each bubble represents the total number of copies in each viral population. The average loop length over time is indicated with a black line. The relative abundance of the three PNG sites was also calculated using a custom script **(bottom)**. Each PNG site is shaded: PNG392 (dark green), PNG397 (blue), and PNG406 (red).

In order to better understand immune pressure on the V4 region we conducted next-generation sequencing of the C3–C5 region at 2, 4, 7, 9, 11, 14, and 29 wpi. We found a decrease in the V4 loop length after the detection of nAbs at 7 wpi: quasispecies with 1–10 residue deletions were present, with 6, 7, and 9-residue deletions (resulting in loop length reduction from 29 to 23, 22, or 20 residues, respectively) the most predominant (Figure 6). Of these deletions, populations that did not have the PNG392 glycan [392Δ6(PNG392-)-like shown in green] were observed at very low levels by 4 wpi (<1%) and increased in frequency up to 11 wpi (20%), after which they decreased rapidly and were found at very low levels again by 29 wpi (2%). While deletions resulted in the removal of PNG392, point mutations introduced PNGs at positions 397 (blue) and 406 (red), both of which mediate neutralization escape (Figure 3C). PNG in positions 397 and 406 were respectively, detected in 8 and 2% at 7 wpi,11 and 39% at 9 wpi, and then decreased to 2.5 and 6.1% by 14 wpi. However, in contrast with the PNG392- mutations, both PNG sites at 397 and 406 were found at considerably higher frequencies (89 and 33%, respectively) by 29 wpi.

These data suggest the CAP63 viral population initially evaded nAb responses by removing parts of the V4 loop, and incorporating PNG sites at positions 397 (to a lesser extent) and 406 (predominantly). However, escape variants where the V4 deletions also resulted in the loss of the PNG at 392 were selected against. Later in infection, the average loop length increased but frequencies of PNG sites at positions 397 substantially increased, suggesting these PNG sites became the predominant mechanism of nAb escape.

### Antibody Escape Mutations Exert Minimal Viral Replicative Fitness Cost in CAP63 Viruses

Deletions in the V4 loop have previously been shown to disrupt Env cell surface display and viral assembly (21), and may therefore impact replicative fitness. Consequently, we sought to determine whether antibody escape mutations in CAP63 were associated with any fitness cost and in particular, whether there was a fitness cost associated with the 392Δ6(PNG392-) mutation, which may account for its lack of persistence.

**Figure 7:**
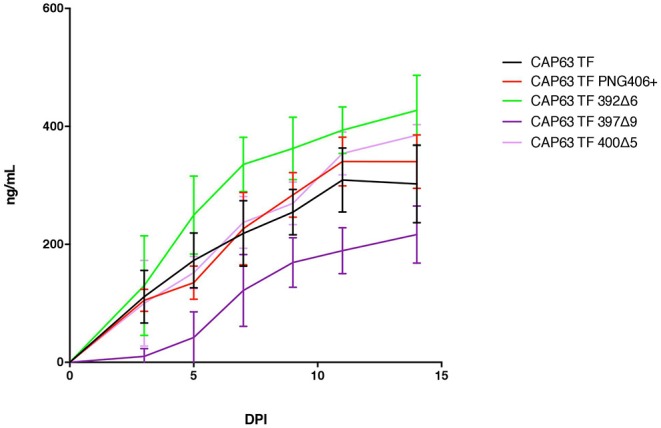
Replication capacity of CAP63 TF and mutant viruses. Average replicative capacities of CAP63 T/F and V4 mutant IMC viruses from 11 weeks post-infection assayed over 14 days in PBMCs. Each data point represents the mean of triplicate wells from two independent experiments.

We evaluated replication of each constructed IMC in PBMCs from two donors over a 14-day period (Figure 7). Overall, replication kinetics of all mutant viruses did not differ appreciably relative to the T/F virus, with the exception of the 397Δ9 virus, which exhibited reduced replicative fitness. Importantly, there was no discernible replicative fitness cost identified in the 392Δ6(PNG392–) mutant virus associated with nAb escape and increased ADCC recognition.

## Discussion

This study showed that viruses with nAb escape mutations that exposed CD4i epitopes resulting in increased ADCC sensitivity, were controlled. ADCC responses in four individuals were detected prior to nAb responses, as shown in other studies (22–24). In one individual (CAP63) who developed potent ADCC and nAb responses to the V4 region, we found that early V4 ADCC responses did not have a noticeable impact on virus sequences, while nAb responses drove rapid escape. However, when nAb escape resulted in a viral variant being more globally susceptible to autologous and heterologous ADCC-mediating antibodies, this escape pathway was blocked. This suggests an indirect role for ADCC responses in controlling viral populations by constraining nAb escape pathways, and potentially a mechanism that keeps HIV in a more ADCC-resistant conformation.

We detected high binding and ADCC responses at enrolment and through early infection, while neutralizing antibodies were detected later in all four participants. This suggests there may be a lower affinity threshold for ADCC-mediating antibodies to bind to the Env and mediate effector functions, while nAbs require further B cell maturation to neutralize virions, which occurs through somatic hypermutation coupled with selection for antigen binding (25). Affinity maturation of nAb lineages has been observed in several longitudinal studies, where binding affinities of the initial antibody to the antigen are substantially lower than subsequent members of a lineage (26, 27).

In addition, in one individual, CAP63, we observed that IgG1 but not IgG3 responses to heterologous gp120 proteins were detected at enrolment, suggesting ADCC responses detected at this time were primarily mediated through IgG1 antibodies, consistent with other studies (28). The magnitude of both of these responses increased substantially around the time nAbs were detected in this individual, although IgG1 responses to a greater extent than IgG3. Both isotypes are known to mediate neutralization and mediate Fc-dependent effector functions including ADCC (17). However, further investigation by isolating monoclonal antibodies from CAP63 will be required to tease apart the role antibody subclasses have had in neutralization and effector functions *in vivo*.

Although ADCC responses were present before autologous nAbs in CAP63, we only detected V4 escape variants concurrent with, or after, the emergence of the nAbs. Most of these nAb escape mutants also facilitated escape from ADCC-mediating antibody responses: five different mutations/deletions observed in CAP63 viruses resulted in resistance to both ADCC-mediating and neutralizing antibodies, providing the first description of the V4 region as a target for ADCC-mediating antibody responses. It is possible that there is an overlap of antibodies or antibody lineages which mediate both functions, which would not be surprising as many nAbs are able to mediate ADCC activity (13, 29).

However, one mutant with a six-residue deletion resulting in the loss of a glycan at 392 [392Δ6(PNG392–)], was resistant to nAbs but highly sensitive to autologous ADCC-mediating antibody responses, as well as a panel of anti-HIV-1 mAbs. Deep sequencing revealed that this mutation was strongly selected against and was found at very low frequency by 29 wpi. Sunshine et al. showed that dynamic escape processes only resolve with the selection of mutations that confer escape with little/no fitness cost to the virus (30). We showed there was no replicative fitness cost incurred by incorporation of the 392Δ6(392NXT–) deletion, suggesting other pressures such as ADCC-mediating antibodies played a role in controlling viral populations through constraining nAb escape pathways.

We previously showed that non-neutralizing antibodies to CD4i epitopes develop to high titers in early infection in the CAPRISA cohort (31). Loss of the PNG392 glycan in CAP63 viruses unmasked a cluster of these epitopes on infected CD4+ cells, as evidenced by the increased sensitivity of this mutant to autologous ADCC responses and CD4i-targeting monoclonal antibodies. Deletions in V4 region have been shown to disrupt gp160 folding (32–34), and consequently may result in exposure of the CD4i cluster of epitopes or other epitopes. ADCC epitopes such as the A32 and C11 clusters, described by Lewis et al. (35), are a major target of potent effector function-mediating non-neutralizing antibodies (34) and have been correlated with decreased disease progression in a recent transmission study (36). These epitopes are often conformationally masked on virion-associated unliganded Env trimers but become exposed upon engagement of gp120 to CD4 and during virus budding, rendering infected cells susceptible to killing by A32- and C11-like antibodies (35, 37).

Importantly, non-neutralizing antibodies targeting these epitope clusters are easily inducible by vaccination and have provided protection in non-human primate models (38). However, a further structural study is required to better understand if the removal of the PNG392 glycan in CAP63 results in the exposure of an entirely novel epitope, or resulted in the unmasking of known epitopes through receptor-dependent conformational changes.

Understanding the interplay between selective forces driving the emergence of viral variants, particularly in early infection, may help identify targets for vaccine design or approaches aimed to eliminate the acute viral reservoir. The limited impact of ADCC-mediating antibodies on viral evolution prior to the detection of nAbs supports the conclusion that there is stronger selective pressure exerted by nAbs compared to ADCC-mediating antibodies. However, ADCC responses may play a supplementary role by limiting nAb escape mutation pathways to those which do not increase susceptibility to non-neutralizing antibody-mediated responses. Here, in one individual we demonstrated a mechanism through which ADCC-mediating antibody responses apply selective pressure by constraining a viable nAb escape pathway. We will continue to study virus-humoral immune responses in early infection in participants of the CAPRISA 002 cohort to define how generalizable these findings are.

## Materials and Methods

### Ethics Statement

Written informed consent was obtained from all participants. This study received ethical approval from the University of the Witwatersrand Human Research Ethics Committee, University of KwaZulu-Natal Biomedical Research Ethics Committee, and University of Cape Town Human Research Ethics Committee. All subjects were adult, and provided written informed consent.

### Study Participant Samples

Samples were obtained from the CAPRISA 002 Acute HIV Infection cohort (Durban, South Africa) (39). Samples were collected at enrolment, weekly for 3 weeks, fortnightly until 3 months and monthly thereafter up to 6 months post-infection. CD4 T-cell counts were assessed using a FACSCalibur flow cytometer, and HIV viral loads were measured using the COBAS AMPLICOR HIV-1 Monitor test, v1.5 (Roche Diagnostics). EDTA blood plasma samples were stored at −70°C until use. Date of infection was estimated as the midpoint between last negative and first positive HIV antibody test or as 14 days prior to a positive RNA result for individuals negative to HIV antibody tests.

### Sequence Analysis

PCR amplification of HIV-1 *env* genes was done using the SGA approach previously described (16, 40) or a limiting dilution PCR. Sequence alignments, amino acid identity plots and participant consensus sequences were generated using BioEdit version 7.0.8.0 (41). The transmitted full-length envelope virus sequence for each participant was taken as the consensus of single genome amplification (SGA) derived sequences (15–42 per participant) generated from the earliest sampled time-point (16). Maximum likelihood trees were generated using Mega version 4 (42). The Los Alamos Highlighter tool (www.hiv.lanl.gov/content/sequence/HIGHLIGHT/highlighter_top.html) was used to generate plots of synonymous and non-synonymous nucleotide changes.

### Viral RNA Extraction and cDNA Synthesis Using the Primer ID (PID) Method

RNA extraction, cDNA synthesis and subsequent amplification were carried out as described previously (43, 44), with the following modifications: cDNA synthesis primers were designed to bind to the C5 (HxB2: 7655-7632) region of the HIV-1 envelope gene. First-round amplification primers were designed to bind to the C3 (HxB2: 7114-7135) region. Raw reads were processed using a custom pipeline housed within the University of Cape Town High Performance Computing core, as previously described (44). The resulting consensus sequences were then used to generate codon aligned nucleotide and amino acid alignments using MACSE (45) and viewed using Bioedit (Version 7.1.11) (41). Amino acid alignments were analyzed for N-glycosylation sites using N-GlycoSite (46), available online at http://www.hiv.lanl.gov/content/sequence/GLYCOSITE/glycosite.html.

### Cloning and Generation of Pseudoviruses

The second round PCR reaction was repeated using the high fidelity Phusion Hot Start DNA Polymerase (Finnzymes), together with, 0.2 mM dNTPs (Roche), 4 μM of *env* 1A-Rx (5′ CAC CGG CTT AGG CAT CTC CTA TAG CAG GAA GAA 3′) and *env*N (5′ CTG CCA ATC AGG GAA AGT AGC CTT GT 3′) in a final volume 50 μL. The amplicons were gel purified and cloned into the mammalian expression vectors pcDNA3.1D/V5-His-TOPO (Invitrogen) or pTarget (Promega, US) according to the manufacturer's instructions. Functional envelope clones were selected using a 96-well plate format pseudovirion entry efficiency assay based on relative luminescence units (RLU) that is 2.5 times above background.

### Cell Lines

TZM-bl cells were obtained from the NIH AIDS Research and Reference Reagent Program Division of AIDS, NIAID, NIH (contributed by Drs. Kappes and Wu). 293T/17 cells were obtained from Dr. George Shaw (University of Pennsylvania, Philadelphia, PA). Both cell lines were cultured in D-MEM (Gibco BRL Life Technologies) containing 10% heat-inactivated fetal bovine serum (FBS) (Biochrom) and 50 μg/mL gentamicin (Sigma). Cell monolayers were disrupted at confluency by treatment with 0.25% trypsin in 1 mM EDTA. CEM.NKR_CCR5_ cells from Dr. Alexandra Trkola were obtained through the NIH AIDS Reagent Program, Division of AIDS, NIAID, and NIH.

### HIV-1 Env Pseudovirus Production and Titration

Stocks of HIV-1 *env* pseudovirus were produced by co-transfecting 293T/17 cells (1.7 × 10^7^ cells per T75 flask) with 4 μg of an HIV-1 *rev/env* expression plasmid and 8 μg of an *env*-deficient HIV-1 backbone plasmid (pSG3ΔEnv) (47) using the PolyFect Transfection Reagent (QIAGEN). Pseudovirus-containing supernatants were harvested 48 h following transfection and clarified by 0.45 μm filtration and adjusted to 20% FBS. Single-use aliquots (1.0 mL) were stored at −80°C. The 50% tissue culture infectious dose (TCID_50_) for each pseudovirus preparation was determined by infection of TZM-bl cells as previously described (31, 47).

### Neutralizing Antibody Assays

Neutralization was measured as described (31) by a reduction in luciferase gene expression after single-round infection of TZM-bl cells with Env-pseudotyped viruses. Titers were calculated as the reciprocal plasma dilution (ID_50_) causing 50% reduction of relative light units (RLU).

### Coating of Cells With Recombinant gp120 HIV-1 Proteins

Recombinant gp120 HIV-1 protein representing the envelope of the HIV-1 subtype C isolate 1086.cΔ7 (GenBank No. DQ435682; Immune Technology, New York, NY, United States), was used to coat CEM.NKR_CCR5_ target cells by incubating 1 × 10^6^ cells in 1 mL RPMI media with 5 μg/mL gp120 for 75 min at 37°C.

### ADCC-GranToxiLux Assay

ADCC activity was detected according to the previously described ADCC-GranToxiLux (GTL) procedure using recombinant gp120 coated CEM. NKR_CCR5_ as target cells and cryopreserved PBMC from a HIV-seronegative donor as effector cells (48). The results of the GTL assay were considered positive if % Granzyme B activity after background subtraction was ≥8% for the infected target cells. The titer of ADCC-mediating antibodies present in the plasma was calculated by interpolating the reciprocal of the last plasma dilution that yielded positive % Granzyme B activity (≥8%).

### HIV-1 Specific Binding Antibody Assay

Plasma HIV-1 specific antibodies to HIV-1 gp120/gp140 proteins and V1/V2 scaffolds were measured by HIV-1 binding antibody multiplex assay for IgG subclasses (IgG1, IgG3) as previously described (11, 23, 49) with the following modifications. Antibody titers (area under the curve, AUC) were determined by serial dilutions of plasma (1:50, 5-fold). IgG subclasses were detected with the following reagents: IgG1 (4E3, Southern Biotech, Birmingham, AL, United States), IgG2 (HP6002, Southern Biotech, Birmingham, AL, United States), IgG3 (HP6050, Southern Biotech, Birmingham, AL, United States), and IgG4 (HP6025, Southern Biotech, Birmingham, AL, United States). The following antigens (provided by Drs. Liao/Haynes, Duke University unless otherwise indicated; Gp41 (Subtype B) (Immunodiagnostics, Scottsdale, AZ, United States); Group M consensus: ConSgp140CFI, Con6gp120/B; Subtype C Envelopes: 1086Cgp140C_avi, C.con.env03 140CF, CAP45_D11gp120.avi/293F, TV1c8_D11gp120.avi/293F; Subtype B Envelope: B.con.env03 140 CF; Subtype A Envelopes: OOMSA4076gp140, A1.con.env03gp140CF_AVI; V1-V2 Antigens: gp70_B.CaseA2 V1/V2/169K, C.1086C_V1_V2 Tags, MulVgp70_His (empty gp70 scaffold), gp70-CAP210.2.00.E8 V1V2, and gp70-CAP 45.2.00.G3 V1V2 (provided by Dr. Abe Pinter, Rutgers University); V2 Antigens: C.1086 V2 tags 293F; Subtype E Envelopes: A244 gp120 gDneg/293F/mon [as described by Yates et al. (50)].

### Construction of Infectious Molecular Clones (IMCs)

An infectious molecular clone representing the transmitted/founder virus sequence of CAP63 was constructed from proviral DNA using the method described in detail by Ochsenbauer et al. (51).

### Mutagenesis of Envelope and Infectious Molecular Clones

Site-directed mutagenesis was used to introduce mutations and deletions into the CAP63_T/F IMC using a QuikChange II site-directed mutagenesis kit (Stratagene) and mutations were confirmed by full length sequencing of the pseudovirus (PSV) or IMC. Point mutations and deletions were named based on HXB2 numbering and number of deleted residues (Δn). Mutations I400T, A410T, 393Δ6, 398Δ7, 397Δ9, and 401Δ5 were introduced into the T/F pseudovirus constructs while A410T, 393Δ6, 397Δ9, and 401Δ5 were introduced into the T/F IMC.

### Generation of Virus Stocks From IMCs

HIV-1 IMC derived virus stocks were produced by transfecting 293T/17 cells (3 × 10^6^ cells per T75 flask) with 12 μg of an HIV-1 IMC plasmid DNA using the FUGENE Transfection Reagent (Roche). Virus-containing supernatant was harvested 48 h following transfection and clarified by centrifugation and 0.45 μm filtration and adjusted to 20% FBS. Single-use aliquots (1.0 mL) were stored at −80°C. The TCID_50_ was determined both by infection of TZM.bl cells and CEM.NKR_LUC_ cells as previously described (31, 47). Twenty five microliters of virus stock was placed in a total volume of 100 μL of GM and 11 serial 5-fold dilutions were made in a 96-well plate. Virus dilutions were co-incubated with 60,000 cells in 100 μl RPMI-12%-GM containing 16 μg DEAE-Dextran/mL for 4 days. On day 4, 100 μl of the cell suspension was placed in a 96-well solid white plate. One hundred microliters of BriteLite Plus (Perkin Elmer) was added to the white plate and incubated for 2 min. Luminescence was read in a luminometer using the 1.0 s/well protocol implemented by the computer program Wallac (Perkin Elmer).

### HIV-1 IMC-Infection of CEM.NKR_****CCR5****_ Cells

HIV-1 IMC virus stocks were titrated to determine the input required for optimal viral gene expression within 72 h post-infection of CEM.NKR_CCR5_ cells as measured by intra-cellular p24 expression. Stocks were used to infect 2 × 10^6^ cells with each IMC by incubation with the appropriate dose for 30 min at 37°C and 5% CO_2_ in the presence of DEAE-Dextran (7.5 μg/mL). The cells were subsequently resuspended at 0.5 × 10^6^/mL and cultured for 2 days in complete medium containing 7.5 μg/mL DEAE-Dextran. On assay day, the infection was monitored by measuring the frequency of cells expressing intracellular p24. The assays performed using the IMC-infected target cells were considered reliable if the percentage of viable p24+ target cells on assay day was ≥20%. Assay data generated using infected cells was normalized to the % of target cells positive for intracellular p24.

### Infected Cell Elimination Assay

HIV-1-infected or mock-infected CEM.NKR_CCR5_ cells were used as targets in the ICE assays and cryo-preserved PBMCs rested overnight in R10 supplemented with 10 ng/ml of IL-15 (Miltenyi Biotec) were used as a source of effector cells. Infected and uninfected target cells were labeled with a fluorescent target-cell marker (TFL4; OncoImmunin) and a viability marker (NFL1; OncoImmunin) for 15 min at 37°C, as specified by manufacturer. Cells were washed in R10 and adjusted to a concentration of 0.2 × 10^6^ cells/mL. PBMCs were then added to target cells at an effector/target ratio of 30:1 (6 × 10^6^ cells/mL). The target/effector cell suspension was plated in V-bottom 96-well plates and co-cultured with 10 μg/mL mAb or 1:100 dilution of plasma. Co-cultures were incubated for 6 h at 37°C in 5% CO_2_.

After the incubation period, cells were washed and stained with anti-CD4-PerCP-Cy5.5 (eBioscience, clone OKT4) at a final dilution of 1:40 in the dark for 20 min at room temperature (RT). Cells were then washed, resuspended in 100 μL/well Cytofix/Cytoperm (BD Biosciences), incubated in the dark for 20 min at 4°C, washed in 1× Cytoperm wash solution (BD Biosciences) and co-incubated with anti-p24 antibody (clone KC57-RD1; Beckman Coulter) to a final dilution of 1:100, and incubated in the dark for 25 min at 4°C. Cells were washed three times with Cytoperm wash solution and resuspended in 125 μL PBS-1% paraformaldehyde. The samples were acquired within 24 h using a BD Fortessa cytometer. The appropriate compensation beads were used to compensate the spill over signal for the four fluorophores. Data analysis was performed using FlowJo 9.6.6 software (TreeStar).

Specific killing was determined by the reduction in % of p24+ cells in the presence of mAbs or plasma after taking into consideration non-specific killing, and was calculated as:

$$\begin{array}{l} \frac{\text{p}24\text{\% }\left(\text{target + effector cells}\right)-\text{p}24\text{\% }\left(\text{targets}+\text{effectors}+\text{mAb}/\text{plasma}\right)}{\text{p}24\text{\% }\left(\text{target}+\text{effector cells}\right)} \end{array}$$

CH65 (an anti-influenza monoclonal antibody) or plasma from a seronegative donor were used as negative controls and A300 (plasma from an HIV-1 chronically-infected individual) was used as a positive control.

### Infected Cell Antibody Binding Assay

Infected CEM.NKR_CCR5_ cells were obtained as described above. Cells incubated in the absence of virus (mock infected) were used as a negative infection control. Following infection, infected and mock infected cells were washed in PBS, dispensed into 96-well V-bottom plates at 2 × 10^5^ cells/well and incubated with 10 μg/mL mAb or 1:100 dilution of plasma for 2 h at 37°C. Subsequently, cells were washed twice with 250 μL/well of wash buffer (1% FBS-PBS; WB), stained with vital dye (Live/Dead Fixable Aqua Dead Cell Stain, Invitrogen) to exclude non-viable cells from subsequent analysis, washed with WB and stained with anti-CD4-PerCP-Cy5.5 (clone OKT4; eBiosciences) to a final dilution of 1:40 in the dark for 20 min at RT. Cells were then resuspended in 100 μL/well Cytofix/Cytoperm (BD Biosciences), incubated in the dark for 20 min at 4°C, washed in 1× Cytoperm wash solution (BD Biosciences) and co-incubated with anti-p24 antibody (clone KC57-RD1; Beckman Coulter) to a final dilution of 1:100 and a secondary FITC-conjugated antibody (goat anti-human IgG(H+L)-FITC, KPL) to a final dilution of 1:100, and incubated in the dark for 25 min at 4°C. Cells were washed three times with Cytoperm wash solution and resuspended in 125 μL PBS-1% paraformaldehyde. The samples were acquired within 24 h using a BD Fortessa cytometer. A minimum of 50,000 total events was acquired for each analysis. Gates were set to include singlet and live events. The appropriate compensation beads were used to compensate the spill over signal for the four fluorophores. Data analysis was performed using FlowJo 9.6.6 software (TreeStar). MFI from wells which included the secondary antibody alone (no mAb or plasma) were subtracted from samples to calculate the MFI specifically due to mAb/plasma binding. CH65 (an anti-influenza monoclonal antibody) or plasma from a seronegative donor were used as negative controls and A300 (plasma from an HIV-1 chronically-infected individual) was used as a positive control.

### Titration of Viral Stocks by β-Gal Staining

The β-Gal reporter found in TZM-bl cells allows direct enumeration of infectious viral units by counting β-Gal expression-positive infected cell colonies under a microscope, as previously described (52). Briefly, 1 × 10^5^ TZM-bl cells in 500 μL of growth media were seeded onto each well of a 24-well plate overnight. On the second day, IMC virus stock was diluted at 1:10, 1:50, 1:250 1:1,250, and 1:6,250 in DMEM containing 1% FBS and antibiotics. Media was removed from the cells and 200 μL of fresh media was added. Fifty microliters of virus dilution and a no-virus control was added to the wells and incubated for 4 h at 37°C. Five hundred microliters of fresh media was added to each well and samples were incubated for 48 h at 37°C. On day 4, supernatant was removed from the infected cells and 250 μL of fixing solution (PBS with 0.8% Gluteraldehyde and 2% formaldehyde) and incubated at room temperature for 8 min. Fixing solution was removed and the cells washed three times with 500 μL PBS per well. Two hundred and fifty microliters of staining solution (4 mM Potassium Ferricyanide, 4 mM Potassium Ferrocyanide, 400 μg/mL MgCl_2_, and 400 μg/mL X-gal in PBS) was added to each well and incubated at 37°C for 2 h. The staining solution was removed and 250 μL PBS added to each well. Blue cells were counted under a light microscope and the number of infectious units calculated. Infectivity was calculated by normalizing the virus stock to 7,500 IU per mL and determining the number of IU per unit p24 determined by ELISA.

### Replication Assay

The kinetics of viral replication was determined in human PBMC. Cells were stimulated for 72 h in RPMI with 15% FCS (Invitrogen) with 2 mM L-glutamine and gentamycin supplemented with 200 U/mL IL2 and phytoheamaglutanin (PHA) at 37°C in 5% CO_2_, as previously described (52). The cells were then washed twice in RPMI with 15% FCS with 2 mM L-glutamine and gentamycin and rested overnight in the same media supplemented with 200 U/mL IL-2 (growth media). A 96-well plate was then seeded with 750,000 PHA-IL2 treated cells per well in 100 μL of growth media. Virus was added to triplicate PBMC wells at a multiplicity of infection (MOI) of 0.01 (MOI, IU/cell) in 100 μL of media. Cells were incubated at 37°C in 5% CO_2_ and 72 μL of culture supernatant was sampled with replacement of media at days 3, 5, 7, 9, 11, and 14 post-infection for determination of p24. Each virus inoculum tested was subjected to the same treatment in the absence of cells to determine background levels of p24. The slope of each growth assay was calculated using the LOGEST function in excel on the logarithmic growth phase of the curve.

### p24 ELISA

COSTAR white opaque 96-well ELISA plates (Corning) were coated overnight with affinity purified sheep anti-HIV-1 p24 gag antibody (Aalto Bio Reagents), washed three times with tris-buffered saline (TBS) and allowed to dry, as previously described (52). Plates were stored at −20°C for medium term use. A p24 standard dilution series was prepared in binding buffer made up of TBS with 1% Empigen (Sigma) after reconstituting bacterially expressed p24 (Aalto Bio Reagents) in TBS with 1% FCS. Virus containing sample supernatants were also serially diluted in binding buffer and 100 μL of samples and p24 standards were placed in pre-coated and blocked plates. Plates were covered and incubated for 2 h at room temperature. The plates were then washed three times with TBS. One hundred microliters of a 1 in 12,500 dilution of an alkaline phosphatase conjugated sheep anti-HIV-1 monoclonal antibody BC 1071-AP (Aalto Biosciences) in TBS with 0.1% TWEEN-20 was added and incubated for 1 h at room temperature. Wells were washed four times with a TBS buffer with TROPIX and 0.1% TWEEN. Bound antibodies were measured by placing 100 μL of a solution of CDP Star with Saphire-II (Life Tech) in TROPIX buffer in each well for half an hour and reading in a luminometer. All reactions were run in triplicate and the equation generated from the linear range of the p24 standard curve was used to determine the amount of p24 in each sample based on the results of the luminometer readings.

## Data Availability Statement

The deep sequencing datasets generated during this study are available in the NCBI short-read archive repository (Accession number: PRJNA586767), Env sequences used have been deposited in the Genbank repository (Accession numbers: MN635324-MN635400).

## Ethics Statement

The studies involving human participants were reviewed and approved by the Human Research Ethics Committee, University of the Witwatersrand; the Biomedical Research Ethics Committee, University of KwaZulu-Natal; and the Human Research Ethics Committee, University of Cape Town. The patients/participants provided their written informed consent to participate in this study.

## Author Contributions

DMi, GB, JP, GF, and CW: conceptualization and methodology. DMi, GB, JP, M-RA, RT, TN, and NY: investigation. NG, CO, JK, GT, DMo, GF, CW, and SK: resources. DMi, GB, and CW: writing – original draft and visualization. PM, LM, GF, and CW: supervision and project administration. DMo, SK, GF, and CW: funding acquisition.

### Conflict of Interest

The authors declare that the research was conducted in the absence of any commercial or financial relationships that could be construed as a potential conflict of interest.

## Supplementary Material

Figure S1 Kinetics of HIV gp120-specific IgG responses in CAP63. Seventeen envelope proteins (gp41/gp140/gp120/V1V2) of various clades and clade consensus sequences and plasma from CAP63 were used in an HIV-1 binding antibody mulitplex assay (HIV-1 BAMA). Binding assay results for IgG1 (left) and IgG3 (right) are calculated as area under the curve (AUC) for each antigen. A panel of 11 gp120/140 proteins of various clades were used to detect gp120-specific IgG1 **(A)** and IgG3 **(B)** binding antibody responses. Gp41-specific IgG1 **(C)** and IgG3 **(D)** binding antibody responses were detected from the first time-point tested (4 wpi). In addition, a panel of five V1, V2, or V1V2 scaffolds of different clades were used to test for the presence of V1V2 IgG1 **(E)** or IgG3 **(F)** binding antibodies.
