# Supplementary figures and images for: Antibody-Dependent Cellular Cytotoxicity (ADCC)-Mediating Antibodies Constrain Neutralizing Antibody Escape Pathway
Source: Front Immunol. 2019 Dec 11;10:2875. doi: 10.3389/fimmu.2019.02875 (PMC6919271; doi:10.3389/fimmu.2019.02875)

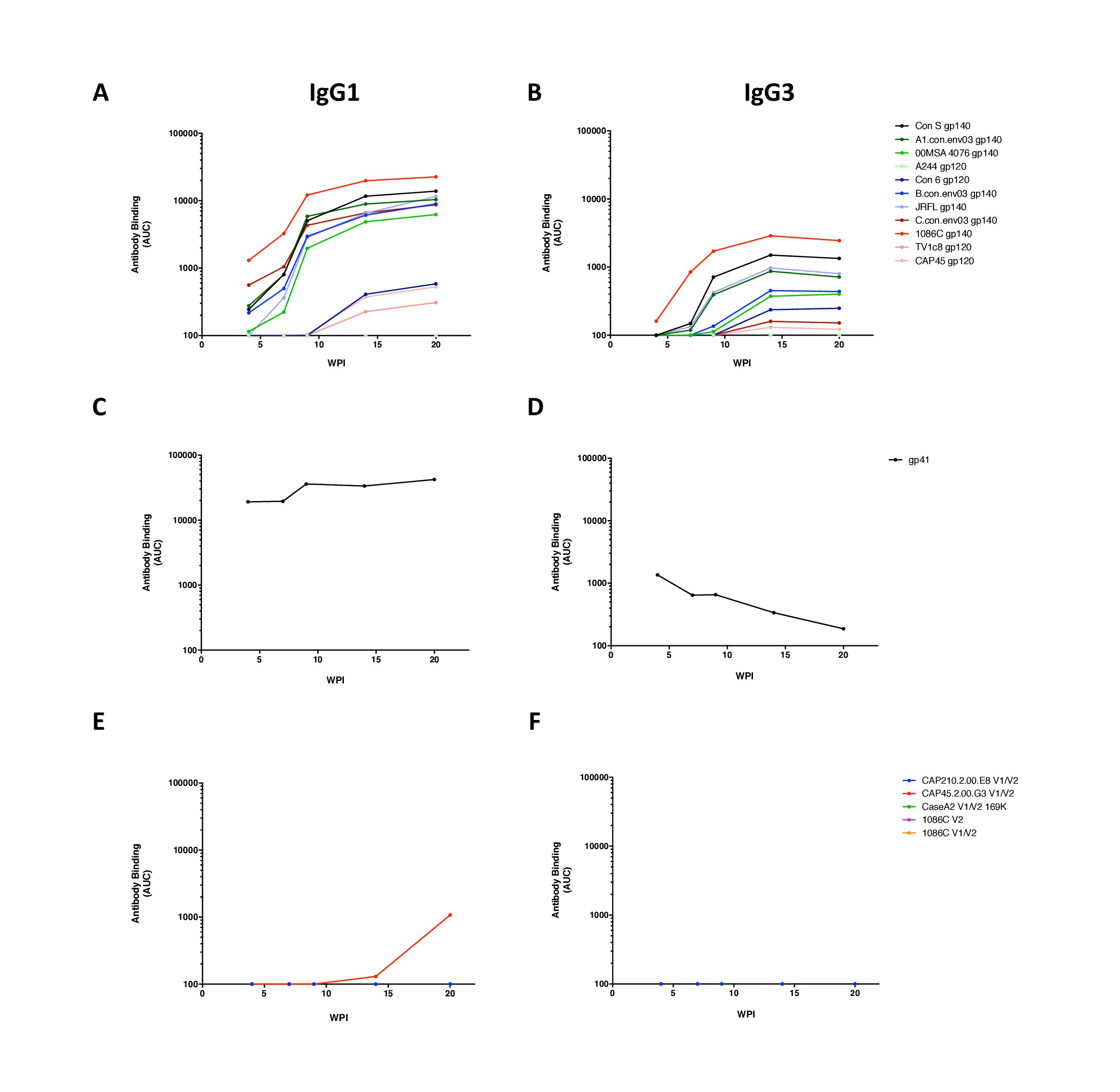

Supplement: Figure S1 — Kinetics of HIV gp120-specific IgG responses in CAP63. Seventeen envelope proteins (gp41/gp140/gp120/V1V2) of various clades and clade consensus sequences and plasma from CAP63 were used in an HIV-1 binding antibody mulitplex assay (HIV-1 BAMA). Binding assay results for IgG1 (left) and IgG3 (right) are calculated as area under the curve (AUC) for each antigen. A panel of 11 gp120/140 proteins of various clades were used to detect gp120-specific IgG1 (A) and IgG3 (B) binding antibody responses. Gp41-specific IgG1 (C) and IgG3 (D) binding antibody responses were detected from the first time-point tested (4 wpi). In addition, a panel of five V1, V2, or V1V2 scaffolds of different clades were used to test for the presence of V1V2 IgG1 (E) or IgG3 (F) binding antibodies. [file Image_1.jpeg]
